# Unusual Large Parosteal Lipoma of the Proximal Forearm: A Case Report and Literature Review

**DOI:** 10.1155/cro/7750483

**Published:** 2025-04-11

**Authors:** Abdullah K. Ghafour, Saywan K. Asaad, Soran S. Raoof, Rezheen J. Rashid, Rebaz M. Ali, Hiwa O. Abdullah, Abdullah A. Qadir, Shvan H. Mohammed, Lawen Jamal Mustafa, Fahmi H. Kakamad

**Affiliations:** ^1^Scientific Affairs Department, Smart Health Tower, Sulaymaniyah, Iraqi Kurdistan, Iraq; ^2^Surgery Department, College of Medicine, University of Sulaimani, Sulaymaniyah, Iraqi Kurdistan, Iraq; ^3^Scientific Affairs Department, Smart Health Tower (Raparin Branch), Sulaymaniyah, Iraqi Kurdistan, Iraq; ^4^Radiology Department, Hiwa Cancer Hospital, Sulaymaniyah, Iraqi Kurdistan, Iraq; ^5^Oncology Department, Hiwa Cancer Hospital, Sulaymaniyah, Iraqi Kurdistan, Iraq; ^6^Kscien Organization for Scientific Research, Sulaymaniyah, Iraqi Kurdistan, Iraq; ^7^Medicine Department, Xzmat Polyclinic, Kalar, Iraqi Kurdistan, Iraq; ^8^Rheumatology Department, Ministry of Health, Sulaymaniyah, Iraqi Kurdistan, Iraq

**Keywords:** forearm mass, Henry's approach, osseous neoplasm, parosteal lipoma, soft tissue tumor, surgical excision

## Abstract

**Introduction:** Parosteal lipomas are rare soft-tissue tumors with challenging surgical management. The current report is aimed at presenting a case of parosteal lipoma in a middle-aged female patient.

**Case Presentation:** A 53-year-old diabetic lady presented with a gradually growing, painless mass in her right proximal forearm for the past 3 years. She complained of fatigue and reduced grip strength. The physical examination indicated a hard, immobile lump. A computed tomography scan revealed a clearly defined fat-density tumor with no bone involvement. Magnetic resonance imaging showed a well-defined fat-density mass surrounding most of the proximal radial shaft. The histological diagnosis of parosteal lipoma was made following surgical excision via Henry's approach.

**Literature Review:** This minireview identified six reports on giant parosteal lipomas, involving patients aged adolescence to 83 years, mainly female. Common complaints were slowly progressive painless swellings. Imaging (radiographs, ultrasounds, CT, and MRI) revealed distinct features of lipomas. Surgical excision was the preferred management, with histopathology confirming lipoma diagnoses. Postoperative outcomes were positive, with no major complications or recurrences reported during follow-up.

**Conclusion:** The tumor is a rare osseous neoplasm that may remain asymptomatic for years until it reaches a size capable of exerting pressure and causing motion difficulty. Meticulous care is paramount during surgical management to prevent iatrogenic nerve injury.

## 1. Introduction

Lipoma is mature adipose tissue's predominant benign soft-tissue tumor [1, 2]. However, osseous lipomas are exceedingly infrequent and are categorized as intraosseous (emerging within bone) or juxtacortical (originating on bone surface). Juxtacortical lipomas can be further classified as parosteal lipomas (PLs) or sub-PLs based on their anatomical connection to the periosteum [3]. It is one of the rarest skeletal neoplasias, comprising less than 0.1% of primary bone tumors and 0.3% of all lipomas [4]. The pathogenesis of ossifying lipoma is under debate [5]. The most prevalent infected sites are the femur, humerus, proximal radius, tibia, clavicle, and pelvis. It predominantly affects adults over 40, regardless of gender [3]. Although there have been no reports of malignant changes, prompt treatment for PL seems necessary before irreversible muscle atrophy occurring, particularly in cases of nerve entrapment. The tumor is typically encapsulated and tightly adhered to the underlying periosteum, especially at sites of osseous growth. This frequently necessitates subperiosteal dissection or segmental bone excision [6].

The current report is aimed at presenting a case of PL in a 53-year-old female patient. The report is organized in line with CaReL criteria and briefly reviews the literature [7].

## 2. Case Presentation

### 2.1. Patient Information

A 53-year-old diabetic lady presented to Smart Health Tower, complaining of a slowly increasing, painless right proximal forearm mass for 3 years. For the last 2 weeks, she has felt easy fatigability and decreased grip strength after a short time of work. She reported no history of trauma.

### 2.2. Clinical Findings

On physical examination, a firm, immobile, painless mass was felt at the anterior proximal forearm, starting just distal to the biceps brachii tendon insertion. The range of motion of the elbow joint and the wrist joint was within normal limits. The distal pulse was positive, and there was no neurological deficit. There was no skin discoloration, scars, or dilated veins.

### 2.3. Diagnostic Approach

**Figure 1:**
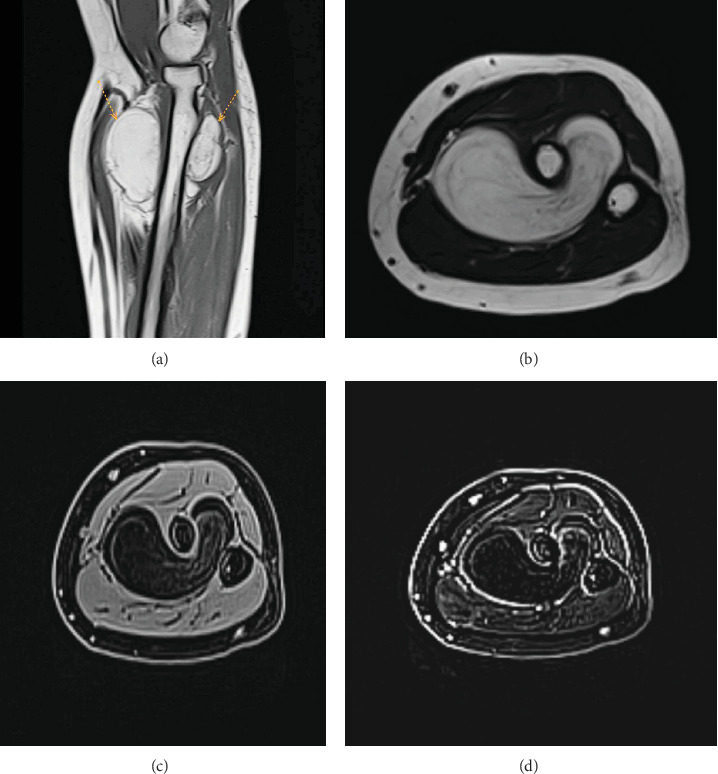
Parosteal lipoma of the proximal forearm is a well-defined lesion at the posteromedial aspect with extension to interosseous space but no intra-articular extension; its closely related to the cortex of proximal radius, with no cortical thinning or underlying bone marrow signal change; the lesion appears as well-defined mass with fat intensity; (a) hyperintense on sagittal T1WI “arrow,” (b) hyperintense on axial T2WI, (c) suppressed of axial fat suppression T1WI, and (d) no appreciable enhancement on axial postprocessed subtraction image of fat suppression T1 pre and postintravenous gadolinium sequences. The lesion is not restricted by diffusion sequence (not shown).

All blood tests, including complete blood count, infection markers, hemoglobin A1C (HbA1C) test, renal function, and viral markers, were within the standard limit. A computed tomography (CT) scan confirmed a well-defined fat-density mass with no bone involvement. A magnetic resonance imaging (MRI) scan showed a large, well-defined 6 × 5.5 × 3.6 cm lobulated fat-intensity space–occupying lesion. The lesion encased more than two-thirds of the proximal radial shaft, extending from the level of the radial neck and tuberosity downward. It surrounds the bone's anterior, medial, and posterior sides, traversing through the intraosseous membrane between the radius and ulna bone space. Additionally, it displaced surrounding vessels without causing intraosseous changes or invading surrounding soft tissues (Figure 1). After giving contrast, no enhancement was seen.

### 2.4. Therapeutic Intervention

The patient underwent surgical excision of the mass through the proximal forearm. By performing Henry's approach, the dissection was made distally between the brachioradialis and flexor carpi radialis muscles. It continued proximally between the brachioradialis muscle and the pronator teres muscle. The superficial radial nerve retracted laterally, whereas the radial artery and its accompanying venae comitantes were medially retracted after separating them from the mass. Later, the supinator muscle was separated from its broad attachment, and the mass was resected in one piece through the incising intraosseous membrane. The mass was sent for histopathological analysis, confirming the diagnosis of PL.

### 2.5. Follow-Up and Outcome

The postoperative period was uneventful, and the neurovascular examination was unremarkable.

## 3. Discussion

The present study performed a minireview of the literature to identify relevant studies on giant PLs. The literature review involved a search to identify relevant studies and was assessed based on specific criteria. A total of six reports on giant PLs were identified (Table 1). The reports spanned different locations and years, with patient ages ranging from adolescence to 83 years old. Both genders were equally affected in the reviewed cases. The presenting complaints included slowly progressive painless swellings, with one case reporting acute and progressive weakness of the right-hand extensors. Clinical examinations revealed well-circumscribed, nontender masses, often with specific characteristics such as being rubbery or immobile. Imaging analyses, including plain radiographs, ultrasounds, CT scans, and MRIs, were frequently used for diagnosis. These imaging techniques revealed various features of the masses, such as soft-tissue shadows, signal intensities indicative of lipomas, and bone-related findings. Surgical excision under general anesthesia was the management of choice in all cases. The histopathological examination (HPE) of the excised masses confirmed the diagnosis of lipoma. Postoperative outcomes were generally favorable, with no major complications or recurrences reported during follow-up.

PLs account for 0.3% of lipomas and 0.1% of primary bone tumors. They are benign soft tissue tumors, but their infiltration into skeletal muscle can resemble malignancies such as liposarcoma [1, 9]. While lipomas predominantly comprise mature adipocytes, rare instances exist where other mesenchymal elements may be present, including smooth muscle, fibrous tissue, cartilage, or bone [5]. The diaphyseal and metaphyseal regions of the long bones are the most commonly affected, and because of their deep placement, this form of tumor tends to be lazy [8]. They were first described in 1836, as reported by Fleming et al. [10]. Patients with PL have ages ranging from 3 months to the eighth decade. Between the ages of 40 and 70, patients made up roughly 50% of the cases [5]. The current case was a 53-year-old diabetic lady patient.

According to the genuine literature, the pathogenesis is still unknown, but three main theories have been outlined [5, 11]. One theory is that these tumors grow as a result of ischemia, metabolic abnormalities, or recurrent trauma, which causes pre-existing fibrous materials inside the lipoma to metaplasia and evolve into osteoblasts. Transforming growth factor (TGF)-*β* may have a role in ossification because it stimulates monocytes to release chemotactic and mitogenic cytokines, which attract mesenchymal and endothelial cells and encourage the creation of collagen and related components of the bone matrix [5].

These lesions typically remain asymptomatic unless complications arise from compression on a nearby neurovascular structure, resulting in neuropraxia, vessel engorgement, or limited mobility of an adjacent joint [2, 9]. The PL manifests as a firm, nontender, gradually enlarging mass over bones without fixing the skin [4]. Murugharaj et al. described a progressively enlarging right forearm mass that was presented for evaluation. The mass caused no neurological deficits but limited elbow flexion due to size and location [12]. Other scholars presented a female case with a history of a painless, progressively enlarging (approximately 4 × 3 × 2 cm) soft mass on the anterolateral aspect of her right proximal forearm. The mass limited terminal pronation [2]. In the current study, the patient presented with a three-year history of a gradually enlarging, painless mass in the right proximal forearm. The examination revealed a firm, immobile, and painless mass in the anterior proximal forearm, just distal to the biceps brachii tendon insertion.

The tumor exhibits characteristic features in imaging modalities. On CT and MRI, it appears as a homogeneous, lobulated mass adherent to the adjacent bone surface. Generally, MRI offers superior evaluation compared to CT. The tumor manifests as a juxtacortical mass with fatty signal intensity on all MRI sequences. Heterogeneity within the lesion is common, reflecting its diverse components. Cartilaginous elements are present in areas with intermediate T1 and high T2 signal intensities. Fibrovascular septa contribute to the lobulated appearance, visualized as low T1 signal intensity strands that become brighter on fat-suppressed images. Moreover, MRI excels at depicting larger areas of bone production within the lipoma. It effectively identifies adjacent muscle atrophy, a consequence of nerve entrapment, by revealing increased fatty striations within the affected muscle, particularly on T2-weighted images. In addition, MRI offers the most accurate depiction of the tumor's relationship with adjacent bone and muscle, which is crucial for surgical planning due to the firm adherence of PL to the underlying cortex at sites of bone production [8]. In the current study, MRI showed a big mass (6 × 5.5 × 3.6 cm) around most of the upper part of the radial bone. It extends from the neck to the lower part of the bone, affecting its front, middle, and back. It breached the intraosseous membrane and displaced surrounding vessels, but no intraosseous involvement or soft tissue invasion was identified. Contrast administration showed no enhancement. A CT scan confirmed it as a clear mass with no bone damage.

Surgical resection represents the definitive treatment for PL. This approach is particularly crucial in cases of nerve entrapment to prevent irreversible muscle atrophy and preserve function. During surgery, meticulous nerve dissection is paramount to avoid iatrogenic nerve injury. Unlike their soft tissue counterparts, PLs often present a surgical challenge due to their firm adherence to the underlying bone. This necessitates a more complex approach compared to the straightforward removal of juxtaposed soft tissue lipomas [4]. To separate the mass from the underlying bone, either subperiosteal dissection or segmental resection may be necessary [8]. Histological examinations often reveal disordered bone and cartilage tissue. Necrosis may or may not be visible. Integral to the diagnosis is identifying mature fatty tissue as the predominant constituent forming the tumor [5]. The mass was successfully excised via Henry's approach with no complications in the present case, and the subsequent HPE confirmed benign PL.

## 4. Conclusion

The tumor is a rare osseous neoplasm that may remain asymptomatic for years until it reaches a size capable of exerting pressure and causing motion difficulty. Meticulous care is paramount during surgical management to prevent iatrogenic nerve injury.

## Figures and Tables

**Table 1:** Summary of six reported cases of giant parosteal lipomas.

**Author/references**	**Places/years**	**Age by years**	**Sex of included patient**	**Presenting compliant**	**Examination**	**Imaging used for diagnosis**		**Management**	**HPE + gross-section**	**Outcome**
						**Plain radiograph + US**	**CT/MRI**			
Alghamdi et al. [1]	Saudi Arabia—2024	54	Female	Slowly progressive, painless swelling of her left arm for 5 years	Rubbery, nontender, well-circumscribed, dome-shaped, mobile mass with elbow motion, measuring 8.5 cm × 4 cm with no LAP	No clear endosteal scalloping of the humeral cortex but tiny cortical radiolucency on the anterior distal humeral cortex. Furthermore, soft-tissue shadows with septations were seen over the distal anterolateral arm	High signal intensity mass that arose from the mid-lateral humerus periosteum and ran within the brachioradialis muscle and nearly covered the entire muscle diameter. Unfortunately, the radial nerve was adherent to the capsule. Furthermore, it showed a classic lipoma signal, which is bright in T1, intermediate in T2, and suppressed in fat	Under GA surgical excision was performed	A lipoma measured 12 cm × 5 cm × 5 cm	Fine in recovery with no wrist or fingers drop

Salama et al. [3]	United Kingdom—2010	83	Female	Acute and progressive weakness of the right-hand extensors with painless swelling in the proximal part of the right forearm	A swelling 3 × 3 cm in the antero-lateral aspect of the right forearm in the region of supinator muscle. The swelling was soft in consistency and decreased in size on flexion with supination of the forearm indicating that the lump was deep to the brachioradialis muscle	Soft-tissue density closely related to the proximal radius with normal bone appearance	Multilobulated mass with hypointensity signal on a T1-weighted sequence which is pathognomonic of a lipoma. The precise anatomical location was in the lateral and anterior aspects of the right radius neck and extending distally for about 3 cm	Under GA surgical excision was performed	A lipoma	The final check, 6 months later, confirmed that elbow, wrist, and hand function were similar in both sides

Chaudhary et al. [4]	India—2013	65	Female	Big mass on the posteromedial aspect of the right upper arm since 1 year	The swelling was slow growing, painless, soft, nontender, immobile, with well-defined margins, and no fixity to skin	There was an evidence of ill-defined soft tissue swelling in the upper arm posteromedially with radiopacity in continuation with the surface of humerus suggestive of bony excrescences	A large 13 cm × 5 cm × 8 cm well defined, nonenhancing, lobulated, heterointense, predominantly fat intensity lesion with a small area of chondroid component measuring 2 cm × 1.6 cm in posteromedial aspect of the proximal right humerus, seen completely separate from the adjacent muscles	Under GA surgical excision by vertical elliptical incision was performed	6 cm × 5 cm × 5 cm sized part of the tumor was under the long head of triceps displacing it medially along with radial nerve and vessels. 7 cm × 6 cm × 3 cm sized part of tumor was under the medial head of triceps displacing it laterally	Was uneventful

Myint et al. [5]	United States—2015	Adolescen	Male	With a 3-year history of a slowly enlarging painless mass alongside his thoracic spine	Was normal, with the exception of a painless, hard, 10 cm mobile mass in the thoracic region. It was cylindrically shaped, smooth, and located longitudinally along the spine. There was no LAP in axillary or groin regions. Musculoskeletal and neurovascular examinations were normal.	An US of the thoracic back showed a homogeneous, discrete solid mass with diffuse calcifications. The mass was excised in whole. It was strongly adherent to the T4, T5, T6 spinous processes, but there was no invasion to the spinal cord.	NA	Under GA a surgical excision was performed	Lipoma	Was uneventful

Xu and Tsang [6]	China—2020	59	Male	Painless swelling in the right upper back	A mild nontender prominence over the right scapula without palpable axillary lymph nodes. Musculoskeletal and neurovascular examinations were normal	A well-circumscribed radiolucent lesion in the body of the right scapula with an adjacent irregular osseous protuberance	CT revealed a well-defined lipomatous subscapular lesion with internal calcific foci adjacent to the body of the scapula with juxtacortical bony excrescence +MRI scan performed for further characterisation showed a well-marginated 3.2-cm × 7.6 − cm × 6.6 − cm soft tissue mass beneath the subscapularis muscle abutting the subscapularis fossa. Signal intensity was predominantly identical in all pulse sequences to that of subcutaneous fat, suggestive of a lipomatous component. The central part of the lesion was heterogeneous in signal intensity, with T1-weighted and T2-weighted low signal structures extending and radiating from the bony scapula, corresponding to the bony protuberance seen on CT scan.	Under local anaesthesia and CT guidance, the right scapular body was penetrated (Hong Kong Med J 2020; 26:70–2 10.12809/hkmj187759) via a posterior approach through the infraspinatus muscle, and the parosteal lipomatous tumour in the subscapular fossa was biopsied multiple times	Tissue mainly consisting of mature fat cells interspersed by thin fibrous septa	Was uneventful

Murugharaji [8]	India—2019	55	Male	With a mass over right forearm for the past 30 years	Swelling was located over proximal half of forearm extending mainly on the anterolateral aspect. Skin overlying it was normal and nonadherent. The mass was soft in consistency and on supination and flexion of forearm muscles; it partially reduced in size indicating that it was in the submuscular plane, specifically deep to the brachioradialis	Spiculated periosteal bone formation at the junction of the proximal and middle one-third junction of the radius. There was an increase in the soft tissue shadow surrounding the lesion, along with characteristic radiodensity of fatty tissue	Multilobulated mass with the hypointense signal on T1-weighted images. The dimension of the mass was estimated to be 8 cm × 8 cm × 4.5 cm. The mass was explored using elliptical incision and it was found underneath brachioradialis muscle	Enbloc resection of the well-capsulated mass with the stalk was carried out	Encapsulated lipoma	Followed-up for 2 years and was found to have a complete range of motion with no radiological or clinical evidence of recurrence of the tumor

## Data Availability

This report's data can be obtained from the corresponding author upon reasonable request.
